# DIALOGUE: Understanding Technology Use in Social Care Delivery

**DOI:** 10.1093/geroni/igaf122.4350

**Published:** 2025-12-31

**Authors:** Hannah Marston, Katie Brittain, Jennifer Lynch, Matthew Lariviere, Raj Mehta, Joanna Thorn, Grant Gibson

**Affiliations:** The Open University, Milton Keynes, England, United Kingdom; Newcastle University, Newcastle, England, United Kingdom; University of Hertfordshire, Hertfordshire, England, United Kingdom; Northumbria University, Newcastle, England, United Kingdom; n/a, n/a, England, United Kingdom; University of Bristol, Bristol, England, United Kingdom; University of Stirling, Stirling, Scotland, United Kingdom

## Abstract

UK social care services for older people, typically provided by local authorities, are struggling to meet demands placed upon them leading to a sense of crisis in the sector. Capitalizing on digital transformation, social care providers are exploring how widely available household smart technologies (e.g., virtual assistants, medication boxes) can be utilized within existing social care services. Underpinned by life course theory, this study explored older adults experiences of, and opinions regarding the continuing and expanding use of technology in social care. Eighteen older (65+) users of social care services were recruited to take part in four co-production workshops in three sites in England (North East, South West and Central England). Workshops employed vignettes of exemplar cases of technology use in social care to generate discussion amongst participants. Descriptive insights were provided offering responses to challenging social care situations encountered by the participants. Findings identified four themes, 1. Current (or everyday) technology use, 2. Benefits of technology use, 3. Concerns with technology use, 4. Key priorities for technology enabled care. Across the four themes, participants noted areas for improvement including clear signposting of information/services provided, in-person care, the want and need to maintain independent living, digital literacy/accessibility concerns, trust in technologies, and training and support. Findings highlight priorities, challenges and concern the ongoing digital transformation of social care in the context of increasing demand and reducing financial resources, for services and their clients. Findings inform educators in gerontology and social work regarding the impacts of technology on social care delivery.

